# Supplementary material for: Genome Wide Association Study of Karnal Bunt Resistance in a Wheat Germplasm Collection from Afghanistan
Source: Int J Mol Sci. 2019 Jun 26;20(13):3124. doi: 10.3390/ijms20133124 (PMC6651844; doi:10.3390/ijms20133124)
Supplement: Supplementary file 1 [file ijms-20-03124-s001.pdf]

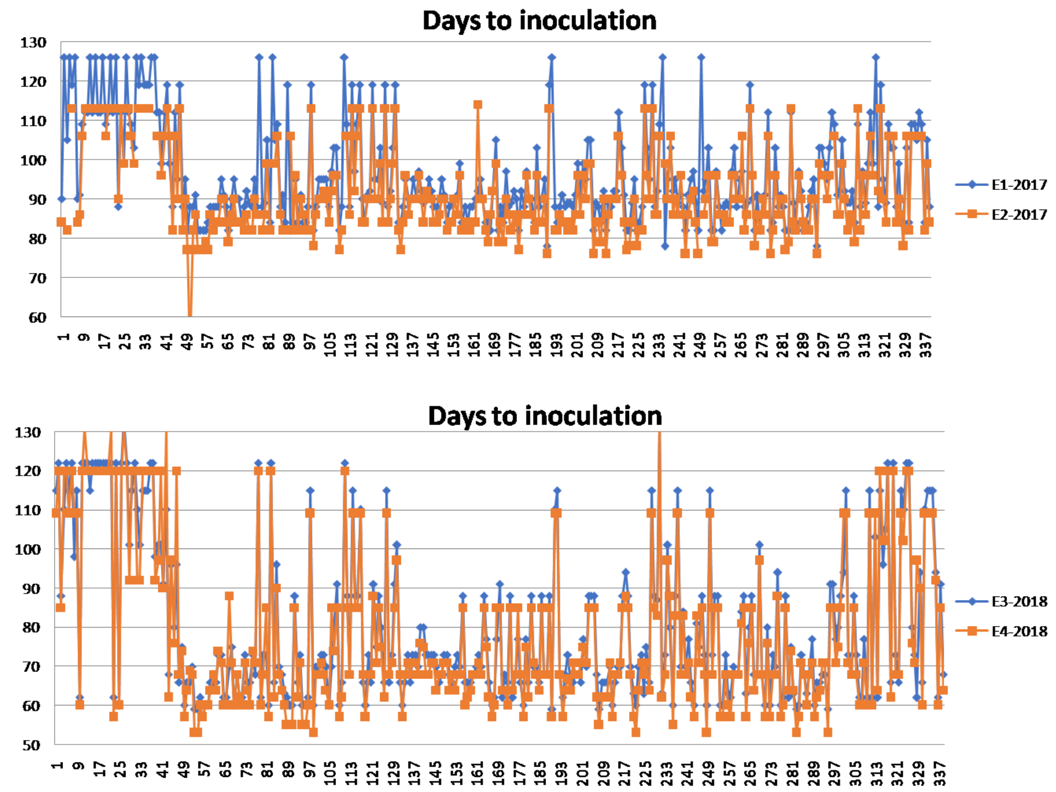

Figure *S*1. Days to inoculation for individual genotypes (a) first and second planting date for year 2016-17 (E1-2017 & E2-2017) and (b) first and second planting date for year 2017-18 (E3-2018 & E4-2018).
